# Chlorate of Potassium in the Treatment of Hemorrhoids

**Published:** 1886-10-20

**Authors:** 


**Chlorate of Potassium in the Treatment of Hemorrhoids.** —The *Therapeutic Gazette* recommends rectal injections of a saturated solution of chlorate of potassium containing ten drops of laudanum. The relief experienced is said to be remarkable. The enemata should be given night and morning.

